# Traumatische Ventrikelseptumruptur nach Explosionstrauma

**DOI:** 10.1007/s00101-023-01307-y

**Published:** 2023-07-05

**Authors:** Gerhard Valicek, Marina Karner, Irmtraut Till, Peter Bergmann, Lena Niederbichler

**Affiliations:** 1 Karl Landsteiner Privatuniversität für Gesundheitswissenschaften, Dr. Karl-Dorrek-Straße 30, 3500 Krems, Österreich; 2 Klinische Abteilung für Anästhesie und Intensivmedizin, Universitätsklinikum St. Pölten, Dunant-Platz 1, 3100 St. Pölten, Österreich; 3 Abteilung für Herzchirurgie, Universitätsklinikum St. Pölten, St. Pölten, Österreich

## Anamnese

Ein 16-jähriger junger Mann erleidet in der Silvesternacht ein Explosionstrauma durch die vorzeitige Detonation eines Böllers (Kugelbombe). Um 0.44 Uhr erfolgt die Schockraumalarmierung mit der Ankündigung: Polytrauma, schweres Schädel-Hirn-Trauma, Amputationsverletzung rechte obere Extremität, offenes Thoraxtrauma. Um 1.40 Uhr wird der Patient vom Team des Notarzthubschraubers Christophorus 2 im Schockraum übergeben. Am Notfallort wurde der Atemweg mittels Endotrachealtubus gesichert, die Beatmung und Oxygenierung erfolgen problemlos. Kreislaufmäßig präsentiert sich der Patient instabil mit notwendigen Bolusgaben von Phenylephrin sowie St.p. CPR im Rahmen einer ventrikulären Tachykardie und einmaliger Defibrillation während des Transports. Der Patient ist mittels Fentanyl, Midazolam, Propofol und Rocuronium narkotisiert. Neurologisch war der Patient am Notfallort primär ansprechbar und trübte dann zusehends ein. Die Untersuchung ergab neben zahlreichen Weichteildefekten eine Amputationsverletzung der rechten oberen Extremität auf Höhe des Handgelenks (mittels Tourniquet versorgt, destruiertes Amputat wird an der Unfallstelle belassen), ein beidseits offenes Thoraxtrauma sowie eine offensichtlich perforierende Verletzung des rechten Auges. Am Notfallort wurden eine Bülau-Drainage links sowie ein Chest-Seal-Verband mit Ventil angelegt.

## Untersuchung

Bei Eintreffen des Patienten im Schockraum um 1.40 Uhr zeigt sich der Patient adäquat ventiliert bei Tachykardie mit verbreiterten Kammerkomplexen und erhöhten ST-Strecken im Monitorbild und weiterbestehender hämodynamischer Instabilität. Die linke Pupille imponiert mittelweit und nicht auf Licht reagibel. Aufgrund der instabilen Situation und der Kreislaufzentralisation erfolgen die Anlage eines dicklumigen zentralen Venenkatheters und die arterielle Kanülierung primär ultraschallgezielt über die rechte Leiste. Im arteriellen Blutgas fallen eine Laktacidose (7,28 mmol/l) und ein nur geringfügig erniedrigter Hämoglobinspiegel von 10,4 g/dl auf.

## Diagnostik

**Figure 1:**
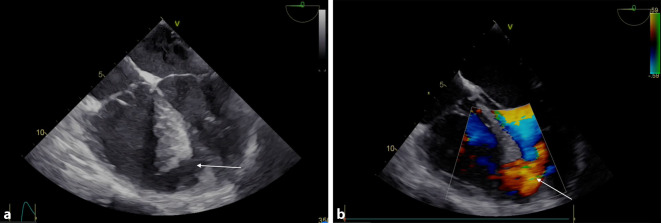

Um 2.02 Uhr wird die Schockraum-CT-Diagnostik durchgeführt, welche einen Hämatopneumothorax links ohne Spannungszeichen bei korrekt liegender Bülau-Drainage ergibt. Weiters finden sich bilateral Lungenkontusionen. Das CCT und Abdomen-CT zeigen einen unauffälligen Befund. Nach der CT-Untersuchung wird der Patient bei systolischem Blutdruckabfall auf 40 mm Hg wieder reanimationsbedürftig (Adrenalingabe und Herzdruckmassage). Aufgrund des thorakalen Verletzungsmusters ist eine echokardiographische Evaluierung lediglich subxiphoidal möglich, die Schallqualität auch von diesem Anlotungspunkt nicht zufriedenstellend. Es kann aber trotzdem (ebenfalls wie in der CT) ein Perikarderguss ausgeschlossen werden. Die Pumpfunktion des linken Ventrikels imponiert gut; der rechte Ventrikel scheint für das jugendliche Alter vergrößert. Daraufhin Durchführen einer transösophagealen Echokardiographie, welche einen unauffälligen Befund der Herzklappen, inklusive der Aorta ascendens, zeigt und neuerlich einen negativen Befund hinsichtlich eines Perikardergusses. Im midösophagealen Vierkammerblick zeigt sich aber apexnah eine Strukturunterbrechung des interventrikulären Septums und mittels Colour-Doppler ein ausgeprägter Links-rechts-Shunt (Abb. 1 und Videos im Zusatzmaterial online).

## Therapie und Verlauf

**Figure 2:**
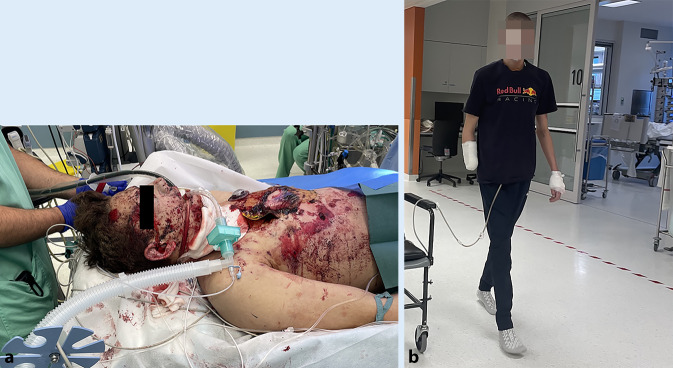

Nach Diagnosestellung einer traumatischen Ventrikelseptumruptur wird der diensthabende Kardiochirurg hinzugezogen (Abb. 2a). Parallel dazu wird durch Rücksprache mit der interventionellen kardiologischen Abteilung die Option einer Akutversorgung der Ventrikelseptumruptur ausgeschlossen. Die hämodynamische Instabilität mit insgesamt 5 Phasen der mechanischen Reanimationspflichtigkeit zwingt zum unmittelbaren Handeln und zur Indikationsstellung einer chirurgischen Sanierung unter extrakorporalem Kreislauf und Vollheparinisierung. Nach Transferierung in den durch Lift unmittelbar mit dem Schockraum verbundenen kardiochirurgischen OP werden primär unter intermittierender CPR die linken Leistengefäße kanüliert, und der extrakorporale Kreislauf wird um 3.40 Uhr begonnen. Danach erfolgen die Thorakotomie und aufgrund inadäquater Blutflüsse über die inguinalen Kanülen eine zentrale Kanülierung. Die chirurgische Sanierung erfolgt über einen rechtsventrikulären Zugang in Form eines Patch aus Rinderperikard, welcher mit Prolen-Einzelkopfnähten und Plegets fixiert wird. Intraoperativ entwickelt der Patient eine weite entrundete lichtstarre Pupille links bei adäquatem Perfusionsdruck, welche sich am Ende der Operation wieder zurückbildet. Nach Öffnen der Aortenklemme weist der Patient biventrikulär eine unzureichende Pumpleistung mit Low-cardiac-output-Syndrom auf, welches aus unserer Sicht multifaktoriell bedingt ist: Sichtbares Hämatom in Vorderwand und Ausflusstrakt des linken Ventrikels, chirurgische Inzision des rechten Ventrikels und generalisiertes myokardiales Kontusionstrauma. Weiters kommt es mehrmals zu massiv blutig schaumiger Sekretion aus dem Endotrachealtubus mit konsekutiver insuffizienter Beatmung. Folglich wird für die postoperative Betreuung eine zentrale venoarterielle ECMO installiert. Im Anschluss erfolgt die unfallchirurgische Versorgung des Amputationsstumpfes und der Weichteilverletzungen sowie augenchirurgisch bei Bulbusruptur die Enukleation des rechten Auges. Die operative Versorgung nimmt insgesamt 10 h in Anspruch, danach wird der Patient intensivmedizinisch betreut.

Am ersten postoperativen Tag wird eine CT-Kontrolle durchgeführt. Erfreulicherweise finden sich keine Zeichen eines hypoxischen Hirnschadens, der maximale NSE-Wert ergibt 27 µg/l (Normwert < 12,5 µg/l). Es folgen tägliche transthorakale echokardiographische Untersuchungen, welche die Dichtigkeit des eingenähten Patch und eine langsame Verbesserung der kardialen Auswurfleistung zeigen. Beatmung und Oxygenierung funktionieren problemlos.

Am 8. postoperativen Tag können die ECMO-Therapie beendet und der Thorax chirurgisch verschlossen werden. Unmittelbar anschließend beginnen die Reduktion der Analgosedierung und das Weaning. Der weitere Verlauf wird noch durch eine septische Phase mit zusätzlicher Rechtsherzdekompensation verkompliziert. Trotzdem kann der Patient am 18. postoperativen Tag bei adäquater Neurologie extubiert werden. Mithilfe der Physiotherapeut*innen legt der Patient bereits am 24. postoperativen Tag einige Schritte in der Intensivstation zurück (Abb. 2b). Neurologisch präsentiert sich der junge Mann völlig unauffällig und kann sich an die Silvesternacht detailliert erinnern. Die Verlegung auf die Normalstation erfolgt am Tag 30 nach Trauma; eine prothetische Versorgung der rechten oberen Extremität ist in Planung.

## Diskussion

Die traumatische Ventrikelseptumruptur nach stumpfem kardialen Trauma ist ein sehr seltenes Ereignis. In der Literatur findet man lediglich vereinzelt Fallberichte. In keiner dieser Kasuistiken waren die Patienten reanimationspflichtig und unmittelbar operativ zu versorgen. Alle Publikationen beschreiben Verläufe nach stumpfem Thoraxtrauma, meist im Rahmen von Verkehrsunfällen bzw. nach Sturz aus großer Höhe. Ein Explosionstrauma als Ursache wurde bisher noch nicht publiziert. Illegale Kugelböller weisen eine Detonationskraft auf, welche in unserem Fall zu einem kriegstypischen Verletzungsmuster führt. Eine Analyse von Obduktionsergebnissen nach stumpfem Thoraxtrauma zeigte in 11,9 % der Fälle ein kardiales Trauma, davon waren nur in 1 % Ventrikelseptumrupturen die Diagnose [2]. Die Mortalität im Fall einer notwendigen Akutversorgung wird mit ca. 50 %, ab einer Defektgröße von 2 cm mit 70 % beschrieben [1].

Lokalisiert sind traumatische Ventrikelseptumrupturen typischerweise im muskulären Anteil apexnah. Die größte Gewalteinwirkung auf das Venrikelseptum durch die Druckwelle entsteht in der isovolumetrischen Kontraktionsphase, in welcher der Druck im linken Ventrikel bereits zum Schluss der Mitralklappe, aber noch nicht zum Öffnen der Aortenklappe führt [1, 3]. Seltener ist der membranöse Anteil des Septums betroffen, dann aber oft in Kombination mit Trikuspidalklappendestruktion. Neben der akuten Ruptur wird in der Literatur auch das zeitlich verzögerte Auftreten im Rahmen konsekutiver Durchblutungsstörung beschrieben.

Die Diagnosestellung ist aufgrund der Rarität herausfordernd. Zusätzlich stoßen fokussierte sonographische Untersuchungsprotokolle, wie eFAST, an ihre Grenzen. In diesen Protokollen stehen der Nachweis eines Perikardergusses und die grobe Beurteilung von Volumenstatus und Pumpfunktion im Vordergrund. Die Größenbeurteilung der Herzkammern mit atypisch vergrößertem rechten Ventrikel hat in diesem Fall aber die transösophageale echokardiographische Untersuchung indiziert. Hämodynamische Instabilität ohne Nachweis eines Perikardergusses nach Thoraxtrauma sollte grundsätzlich in einer strukturierten Echokardiographie münden, sofern ein hämorrhagischer Schock die Symptomatik nicht eindeutig erklärt [5]. Aber auch die TEE-Diagnostik birgt Fallstricke. Die Diagnostik bei apexnaher Lokalisation setzt voraus, dass der Schallsektor den Apex des linken Ventrikels erfasst. Dazu ist im midösophagealen Vierkammerblick eine Retroflexion der Schallsonde notwendig. Bei Foreshortening des linken Ventrikels ist die Pathologie nicht sichtbar. In sämtlichen Kasuistiken wird auskultatorisch ein ausgeprägtes Systolikum beschrieben, welches hinweisgebend sein kann. Computertomographisch ist der Ventrikelseptumdefekt auch diagnostizierbar, wurde in unserem Fall aber erst mit deutlicher zeitlicher Latenz nach eigener Diagnosestellung festgestellt.

Therapeutisch ist die verzögerte chirurgische Versorgung die Therapie der Wahl, bei kleinen Defekten mit geringem Shunt-Volumen ist auch ein konservatives Prozedere möglich [4]. In diesem Fall führt die höchstgradige hämodynamische Instabilität zur unmittelbaren chirurgischen Versorgung. Als Bridging kann auch die Anlage einer intraaortalen Ballonpumpe indiziert sein, um die linksventrikuläre Nachlast zu senken und das Shunt-Volumen zu reduzieren. Lässt sich damit keine Stabilisierung erreichen, ist die venoarterielle ECMO die einzige Option als Überbrückung bis zur chirurgischen Versorgung [4].

## Fazit für die Praxis

Rupturierende kardiale Verletzungen nach stumpfem Trauma sind selten und mit hoher Mortalität assoziiert. Hämodynamische Instabilität ohne Nachweis eines Perikardergusses muss zu einer vollständigen echokardiographischen Evaluierung führen, da sonographische Point-of-care-Untersuchungsprotokolle in diesem Fall unzureichende Informationen liefern. Verletzungsmuster dieser Schwere und Seltenheit können nur durch perfektes Funktionieren aller multiprofessionellen und interdisziplinären Glieder der Versorgungskette überlebt werden. Dieser Fall bestätigt das Prinzip aus dem ATLS, dass der richtige Patient das richtige Versorgungszentrum zum richtigen Zeitpunkt erreichen muss.

## Supplementary Information







## Abkürzungen

ATLS: Advanced Trauma Life Support
CCT: Zerebrale Computertomographie
CPR: Kardiopulmonale Reanimation
CT: Computertomographie
ECMO: Extrakorporale Membranoxygenierung
eFAST: Extended focused assessment with sonography for trauma
NSE: Neuronenspezifische Enolase
POCUS: Point of care ultrasound
TEE: Transösophageale Echokardiographie
