# Supplementary figures and images for: Traumatic rupture of the ventricular septum after blast trauma
Source: Anaesthesiologie. 2023 Jul 5;72(8):580–3. [Article in German] doi: 10.1007/s00101-023-01307-y (PMC10400476; doi:10.1007/s00101-023-01307-y)

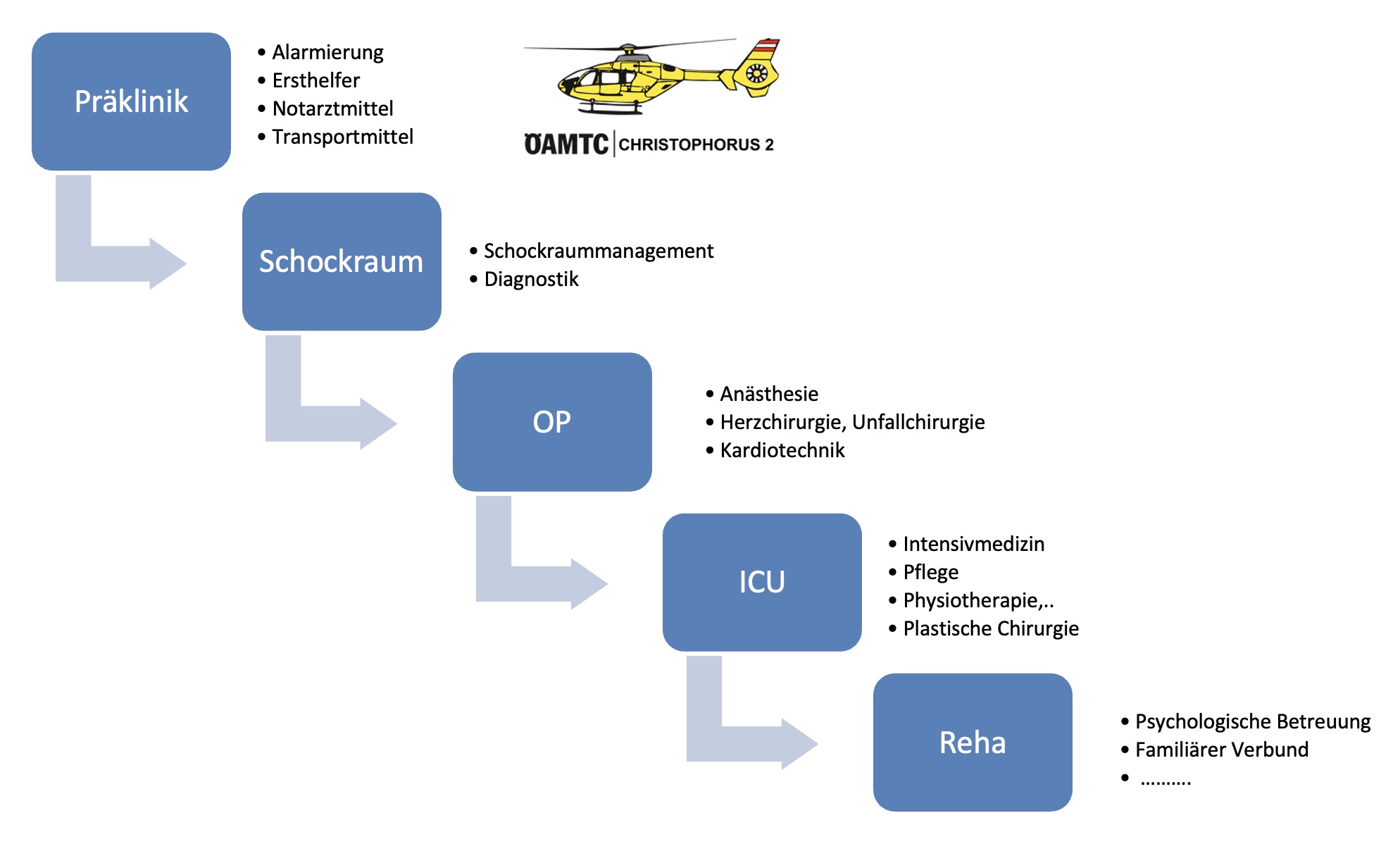

Supplement: Supplementary file 1 [file 101_2023_1307_MOESM1_ESM.jpg]
